# Clazakizumab for the treatment of chronic active antibody-mediated rejection (AMR) in kidney transplant recipients: Phase 3 IMAGINE study rationale and design

**DOI:** 10.1186/s13063-022-06897-3

**Published:** 2022-12-22

**Authors:** Peter W. Nickerson, Georg A. Böhmig, Steve Chadban, Deepali Kumar, Roslyn B. Mannon, Teun van Gelder, James C. Lee, Scott Adler, Edward Chong, Arjang Djamali

**Affiliations:** 1 University of Manitoba, Winnipeg, MB Canada; 2 Medical University of Vienna, Vienna, Austria; 3 University of Sydney, Sydney, Australia; 4 University of Toronto, Toronto, ON Canada; 5 University of Nebraska Medical Center, Omaha, NE USA; 6 Leiden University Medical Center, Leiden, Netherlands; 7 CSL Behring, 1020 First Ave, King of Prussia, PA USA; 8 University of Wisconsin, Madison, WI USA

**Keywords:** Chronic active antibody-mediated rejection, Clazakizumab, Estimated glomerular filtration rate, Kidney transplantation

## Abstract

**Background:**

Chronic active antibody-mediated rejection (AMR) is a major cause of graft loss with no approved drugs for its treatment. Currently, off-label regimens are used, reflecting the high unmet need for effective therapies based on well-controlled trials. Clazakizumab is a high-affinity, humanized monoclonal antibody that binds interleukin-6 and decreases donor-specific antibody (DSA) production and inflammation. Phase 2 pilot studies of clazakizumab in kidney transplant recipients with chronic active AMR suggest modulation of DSA, stabilization of glomerular filtration rate (GFR), and a manageable safety profile. We report the design of the Phase 3 IMAGINE study (NCT03744910) to evaluate the safety and efficacy of clazakizumab for the treatment of chronic active AMR.

**Methods:**

IMAGINE is a multicenter, double-blind trial of approximately 350 kidney transplant recipients with chronic active AMR (Banff chronic glomerulopathy [cg] >0 with concurrent positive human leukocyte antigen DSA) randomized 1:1 to receive clazakizumab or placebo (12.5 mg subcutaneous once every 4 weeks). The event-driven trial design will follow patients until 221 occurrences of all-cause graft loss are observed, defined as return to dialysis, graft nephrectomy, re-transplantation, estimated GFR (eGFR) <15 mL/min/1.73m^2^, or death from any cause. A surrogate for graft loss (eGFR slope) will be assessed at 1 year based on prior modeling validation. Secondary endpoints will include measures of pharmacokinetics/pharmacodynamics. Recruitment is ongoing across North America, Europe, Asia, and Australia.

**Discussion:**

IMAGINE represents the first Phase 3 clinical trial investigating the safety and efficacy of clazakizumab in kidney transplant recipients with chronic active AMR, and the largest placebo-controlled trial in this patient population. This trial includes prognostic biomarker enrichment and uniquely utilizes the eGFR slope at 1 year as a surrogate endpoint for graft loss, which may accelerate the approval of a novel therapy for patients at risk of graft loss. The findings of this study will be fundamental in helping to address the unmet need for novel therapies for chronic active AMR.

**Trial registration:**

ClinicalTrials.govNCT03744910. Registered on November 19, 2018.

**Supplementary Information:**

The online version contains supplementary material available at 10.1186/s13063-022-06897-3.

## Background

Chronic active antibody-mediated rejection (AMR) is a leading cause of graft failure in kidney transplant recipients [1, 2]. The salient features of active AMR include acute tissue injury, antibody interaction with vascular endothelium, and the presence of circulating donor-specific antibodies (DSA), with chronic active AMR diagnosed using additional evidence of chronic tissue injury [3]. The continuum of injury and inflammation produced by chronic active AMR manifests as several morphologic features of ongoing injury, including severe peritubular capillary basement membrane multilayering and transplant glomerulopathy (TG), and a progressive decline in renal function on the pathway to graft loss [1, 3, 4]. Patients who experience graft loss are required to return to dialysis and wait for re-transplantation, both of which have a significant impact on mortality [5, 6].

Presently, no therapies are approved for chronic active AMR, and the current standard of care is based on inconclusive data from small, poorly controlled studies [2]. Off-label regimens have been recommended by expert groups for the treatment of chronic active AMR; these regimens largely focus on the optimization of baseline immunosuppression [2]. As such, there is a high unmet need for effective therapies for chronic active AMR utilizing robust data from randomized controlled trials [2].

Interleukin (IL)-6 is a pleiotropic cytokine that mediates inflammation and modulates the immune response [7, 8]. Murine studies of AMR have suggested a role for IL-6 in driving B-cell activation and differentiation to antibody-producing plasma cells, which can damage a graft [9–12]. Preliminary murine data have indicated that IL-6 may also inhibit immune regulatory T (T_reg_) cells and their associated promotion of graft tolerance [10]. In humans, post-transplant studies of kidney recipients have reported elevated IL-6 levels in serum and urine prior to transplant rejection episodes [13, 14].

Inhibition of IL-6 signaling has been evaluated as a therapeutic approach in chronic AMR. Tocilizumab, an anti-IL-6 receptor (IL-6R) monoclonal antibody, is approved in a range of therapeutic areas, including the treatment of rheumatoid arthritis and juvenile idiopathic arthritis [15, 16]. Tocilizumab has been used off label to treat AMR in small studies, driving significant reductions in DSA, stabilization of renal function, and improvement in various Banff inflammation scores, though results varied by study [15, 17, 18]. In one study of 36 patients with chronic active AMR, graft and patient survival rates following 2 years of tocilizumab treatment were 80 and 91%, respectively, 6 years post-diagnosis [15]. While this provides evidence that blocking the IL-6 pathway conveys clinical benefit for AMR patients, the ability to draw meaningful conclusions from these studies is limited by their small sample size, lack of randomization, and poorly controlled study design. Clazakizumab is a genetically engineered, high-affinity, humanized monoclonal antibody that binds IL-6, thereby decreasing DSA production and inflammation, and slowing the decline in the estimated glomerular filtration rate (eGFR) [19–21]. Clazakizumab has demonstrated an acceptable safety profile when evaluated in other IL-6-related conditions such as rheumatoid arthritis and psoriatic arthritis [22, 23]; however, clazakizumab has not yet been approved for any indication. Clazakizumab has recently been investigated in kidney transplant recipients in both the pre-transplant setting as part of a desensitizing regimen [9, 19, 24–26] and the post-transplant setting for the treatment of AMR; results from these studies have provided proof-of-concept results supporting further clinical development of clazakizumab [27–29].

Clazakizumab is the most potent and longest-acting agent in the IL-6/IL-6R-blocking category that shows promise in the management of kidney transplant recipients [10, 30]; it binds IL-6 and blocks both classical signaling via membrane-bound IL-6R and trans-signaling via soluble IL-6R [10]. Pharmacokinetic (PK) data demonstrate that clazakizumab has a half-life of approximately 30 days and monthly subcutaneous (SC) injections allow for sustained IL-6 blockade [10, 30]. In addition, clazakizumab lacks both antibody-dependent cellular and complement-dependent cytotoxicity and also does not crosslink any surface receptors [30]. A small Phase 2 trial has suggested that clazakizumab was not associated with the potentially harmful rebound phenomenon due to IL-6 accumulation that has been observed with other methods of IL-6R blockade, such as tocilizumab [15, 29].

Here, we report the design of the Phase 3 *I*nterleukin-6 Blockade *M*odifying *A*ntibody-mediated *G*raft *In*jury and *E*stimated Glomerular Filtration Rate Decline (IMAGINE) study (NCT03744910) to evaluate the safety and efficacy of clazakizumab for the treatment of chronic active AMR in kidney transplant recipients, a multinational trial which has initiated recruitment.

## Methods/design

### Study objectives

The primary objectives of the IMAGINE study are to evaluate the safety and efficacy of clazakizumab in preventing all-cause, composite graft loss (defined as sustained return to dialysis, graft nephrectomy, re-transplantation, a sustained eGFR <15 mL/min/1.73 m^2^, or death from any cause) and slowing/preventing the progressive loss of kidney function (as measured by eGFR) due to chronic active AMR. The primary efficacy endpoint is time to all-cause composite graft loss.

Secondary objectives are to further evaluate the efficacy of clazakizumab on time to death-censored graft loss; albuminuria; DSA titers and mean fluorescence intensity (MFI) scores; incidence of acute rejection episodes (T-cell-mediated rejection [TCMR] and AMR); histology of kidney biopsies according to the Banff 2015 lesion grading scores [31]; overall patient survival; and the PK properties and immunogenicity of clazakizumab in kidney transplant recipients with chronic active AMR.

Exploratory objectives are to evaluate the pharmacodynamics (PD) of clazakizumab (serum IL-6 [total and free] and/or high-sensitivity C-reactive protein [hsCRP]) following SC injection; to explore the relationship between clazakizumab PK and PD; and to evaluate the effects of clazakizumab on healthcare utilization and health-related quality of life due to chronic active AMR in kidney transplant recipients.

### Inclusion criteria

The target population of the IMAGINE study is kidney transplant recipients aged 18–70 years with chronic active AMR. Patients are required to have received a kidney transplant from a living or deceased donor at least 6 months prior to screening. Diagnosis of chronic active AMR is determined by the presence of post-transplantation HLA DSA, using single-antigen bead-based assays, and specific histopathologic diagnostic criteria based on the Banff 2015 criteria [31], which will be reviewed by the central HLA reviewer and central pathologist, respectively. Select inclusion criteria for the study are summarized in Table 1.

**Table 1:** Select inclusion and exclusion criteria

**Inclusion criteria**	
1. Living donor/deceased donor kidney transplant recipients ≥6 months from time of transplant 2. Diagnosis of chronic active AMR determined by kidney biopsy and the presence of HLA DSA using single-antigen bead-based assays. The following histopathologic and serologic diagnostic criteria (based on Banff 2015 criteria [31] must be met for inclusion: • Morphologic evidence of chronic tissue injury, as demonstrated by TG (cg>0). Biopsies without evidence of chronic tissue injury on light microscopy, but with glomerular basement membrane double contours on electron microscopy (cg1a) are eligible • Evidence of current/recent antibody interaction with vascular endothelium • Serologic evidence of circulating HLA DSA	
**Key exclusion criteria**	
1. eGFR <25 mL/min/1.73 m^2^ or >65 mL/min/1.73 m^2^ (MDRD4) 2. Nephrotic range proteinuria defined as spot UACR ≥2200 mg/g (≥220 mg/mmol). If spot UACR is above defined limits, a single repeat test can be performed on a separate day to confirm ineligibility 3. Multiorgan transplant recipient (except for simultaneous kidney–pancreas or previous multiple kidney transplants) or cell transplant (islet, bone marrow, stem cell) recipient 4. Treatment for AMR (including chronic active AMR) or TCMR within 3 months of the start of screening 5. Received T-cell-depleting agents (e.g., alemtuzumab or anti-thymocyte globulin) within 3 months of the start of screening 6. Treatment with mTOR inhibitors within 4 weeks of the start of screening 7. Biopsy indicating predominant cause of renal dysfunction caused by pathology other than chronic active AMR 8. Impaired renal function due to disorders in the transplanted graft (e.g., renal artery stenosis or hydronephrosis) 9. Neutropenia (<1000/mm^3^) or thrombocytopenia (<50,000/mm^3^) 10. Prior exposure to clazakizumab, tocilizumab, or other IL-6/IL-6R blockers 11. ABO-incompatible transplant recipient 12. Severe hypogammaglobulinemia (defined as IgG <400 mg/dL) 13. Prior (within 2 years of the start of screening) exposure to proteasome inhibitors (e.g., bortezomib)	

The full list of criteria can be found at clinicaltrials.gov

*AMR* antibody-mediated rejection, *cg* chronic glomerulopathy, *DSA* donor-specific antibodies, *eGFR* estimated glomerular fibrillation rate, *HLA* human leukocyte antigen, *IgG* immunoglobulin G, *IL-6* interleukin-6, *IL-6R* IL-6 receptor, *MDRD4* Modification of Diet in Renal Disease 4-variable version, *mTOR* mammalian target of rapamycin, *TCMR* T-cell-mediated rejection, *TG* transplant glomerulopathy, *UACR* urine albumin-to-creatinine ratio

### Exclusion criteria

Key exclusion criteria include prior multiorgan transplant (except for simultaneous kidney–pancreas or previous multiple kidney transplants) or cell transplantation (islet, bone marrow, or stem cell); treatment of AMR or use of T-cell-depleting agents within 3 months of screening; eGFR <25 mL/min/1.73 m^2^ or >65 mL/min/1.73 m^2^; and nephrotic range proteinuria, defined as spot urine albumin-to-creatinine ratio (UACR) ≥2200 mg/g. The eGFR cutoff range has been included in the exclusion criteria. Specifically, the lower eGFR cutoff point was chosen to avoid recruiting recipients with advanced renal failure that is likely to be irreversible, while the higher eGFR cutoff point was chosen to include a subset of the study population who were more likely to inform on the utility of clazakizumab in the treatment of chronic active AMR within the 5-year study period [4, 32]; the proteinuria cutoff was selected because substantially elevated levels of urinary protein are associated with a worse graft outcome [33, 34]. Pregnancy monitoring will be carried out at screening and prior to every dose during the trial. Pregnant patients will permanently discontinue with the investigational product and continue in the trial per protocol. Select exclusion criteria for the study are summarized in Table 1.

### Study design

**Fig. 1:**
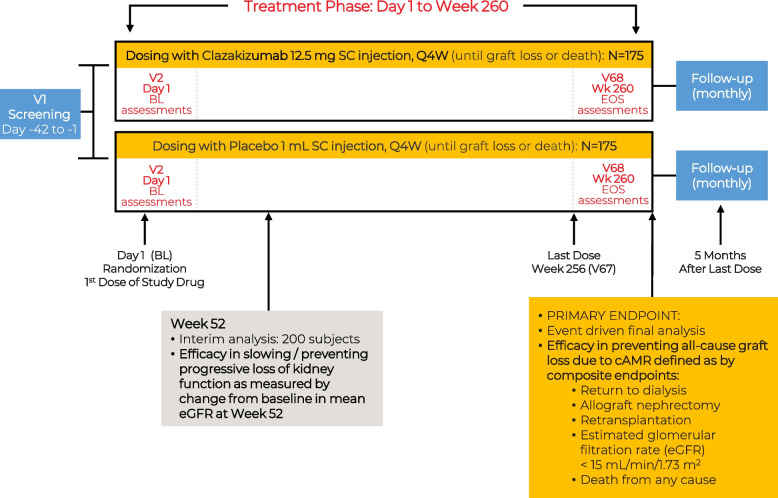
IMAGINE study design. BL, baseline; cAMR, chronic active antibody-mediated rejection; EOS, end of study; eGFR, estimated glomerular filtration rate; Q4W, once every 4 weeks; SC, subcutaneous

**Fig. 2:**
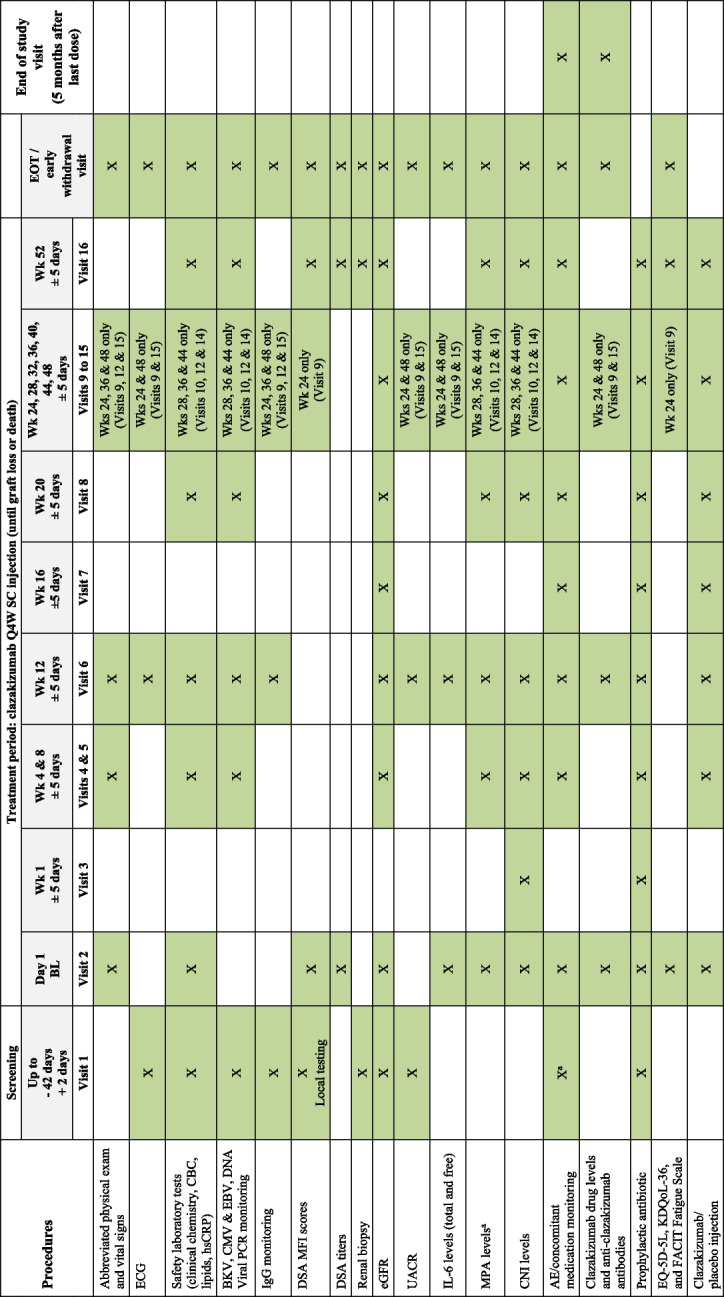
IMAGINE study schedule of events for year 1. ^a^AEs occurring during the Screening Period are to be recorded as Medical History. Any AE that meets the definition of a SAE must also be reported to CSL Behring (or its delegate: e.g., CRO) within 24 h of site awareness. AE, adverse event; BKV, polyoma BK virus; BL, baseline; CBC, complete blood count; CMV, cytomegalovirus; CNI, calcineurin inhibitor; DSA, donor-specific antibodies; EBV, Epstein–Barr virus; ECG, electrocardiogram; eGFR, estimated glomerular fibrillation rate; EOT, end of treatment; EQ-5D-5L, EuroQOL-5 dimensions, 5 levels questionnaire; FACIT, Functional Assessment of Chronic Illness Therapy; hsCRP, high-sensitivity C-reactive protein; IgG, immunoglobulin G; IL-6, interleukin-6; KDQoL-36, Kidney Disease Quality of Life 36-Item Short-Form Survey; MFI, mean fluorescence intensity; MMF, mycophenolate mofetil; MPA, mycophenolic acid; PCR, polymerase chain reaction; Q4W; once every 4 weeks; SAE, serious AE; SC, subcutaneous; UACR, urine albumin-to-creatinine ratio; wk, week

IMAGINE is a randomized, double-blind, parallel-group, placebo-controlled, Phase 3 multicenter superiority study of kidney transplant recipients with chronic active AMR (Fig. 1). The key scheduled events are outlined in Fig. 2, and the protocol will be reported following the SPIRIT guidelines (Additional file 1). Patients will be screened up to 6 weeks prior to treatment at visit 1, and approximately 350 patients who satisfy the inclusion and exclusion criteria will be randomized 1:1 to receive clazakizumab (12.5 mg) or placebo (0.9% weight per volume NaCl as a sterile solution) by SC injection every 4 weeks (Q4W) until 221 episodes of graft loss have occurred. To ensure the integrity of blinding, all patients will receive placebo via SC injection, including those not receiving SC delivery of standard of care. Randomization will be done centrally via an Interactive Response Technology (IRT) using a stratified block randomization scheme; only unblinded roles in the IRT system will have access to treatment assignment to maintain the blinding. Stratification factors will include baseline eGFR (25–45 mL/min/1.73 m^2^ or >45–65 mL/min/1.73 m^2^); baseline proteinuria (UACR <300 mg/g [<30 mg/mmol] or UACR ≥300 mg/g [≥30 mg/mmol]); treatment for early (within 6 months of transplant) AMR rejection episodes (yes/no); and treatment for late (more than 6 months post-transplant) AMR rejection episodes (yes/no).

Baseline assessments will be performed before the first dose of investigational drug at day 1 (visit 2). Safety monitoring will be conducted prior to dosing, and a post-treatment follow-up will be carried out monthly for 5 months after the last treatment dose to monitor adverse events (AEs) and pregnancies.

### Other interventions

Treatments for the continuum of AMR and TCMR are not permitted within 3 months of the start of screening. Patients who received these treatments at any time prior to that must have both a renal biopsy and DSA testing performed after halting/completing treatment to confirm eligibility. The following treatments are prohibited for the duration of the study: anti-IL-6/IL-6R monoclonal antibodies, eculizumab, intravenous immunoglobulin (IVIg; except for treatment of hypogammaglobulinemia during the study), mammalian target of rapamycin inhibitors (everolimus, sirolimus), plasma exchange (PLEX), proteasome inhibitors, rituximab, and T-cell-depleting agents.

Permitted concomitant medications include anti-diabetic agents, anti-hypertensive agents (e.g., angiotensin-converting enzyme inhibitors [ACEIs] or angiotensin II receptor blockers [ARBs]), azathioprine (recommended 1.0–2.0 mg/kg/day), belatacept (only if treatment was initiated prior to screening), calcineurin inhibitors (target plasma trough levels of 50–150 ng/mL for cyclosporine and 5–8 ng/mL for tacrolimus), low-dose corticosteroids (prednisone/prednisolone ≤10 mg/day), and mycophenolate mofetil (MMF; 1.0–1.5 g/day)/mycophenolic acid (720–1080 mg/day). If a patient is already on an ACEI or ARB, or is starting these drugs, the dose should be stabilized for at least 2 months prior to the screening visit.

All patients will be required to take prophylactic treatment for *Pneumocystis jiroveci* pneumonia, which will consist of 80 mg of trimethoprim daily or 160 mg of trimethoprim 3 times per week during year 1 (or an alternative acceptable agent if already receiving prior to starting the study), and per the discretion of the investigator for years 2 to 5 while participating in the study. Treatment of acute TCMR with pulse steroid is permitted, as is treatment of cytomegalovirus (CMV) infection with oral valganciclovir or intravenous ganciclovir.

### Study assessment and endpoint analyses

The primary endpoint will be analyzed on an intention-to-treat (ITT) basis as time to all-cause graft loss (defined as a sustained return to dialysis, graft nephrectomy, re-transplantation, a sustained eGFR <15 mL/min/1.73 m^2^, or death from any cause); the event-driven endpoint will be analyzed when at least 221 all-cause graft loss events have occurred over 5 years (Table 2). Based on modeling validation in kidney transplant recipients with late AMR, this is the first study of chronic AMR treatment that utilizes the eGFR slope as a surrogate endpoint to serve as a predictor for all-cause graft loss. As such, an interim efficacy analysis of difference in eGFR slope between treatment groups will be assessed when approximately 200 patients completed 1 year of follow-up (Interim Analysis 2) [4]. A sample size re-estimation for Interim Analysis 2 will be conducted when approximately 100 patients have completed 1 year of follow-up (Interim Analysis 1). A PK/PD sub-study will be performed on a target of 44 patients; the endpoints are outlined in Table 2. Blood samples will be collected for assessment of clazakizumab PK in serum and for assessment of PD biomarkers, including but not limited to plasma IL-6 levels (total and free) and/or serum hsCRP.

**Table 2:** Primary and secondary endpoints

**Primary endpoint**	
Time to all-cause graft loss defined as any of: • Return to dialysis • Graft nephrectomy • Re-transplantation • eGFR <15 mL/min/1.73m^2^ • Death from any cause This event-driven endpoint will be analyzed when at least 221 all-cause graft loss events have occurred	
**Interim efficacy analysis**	
• Interim Analysis 1: sample size re-estimation when approximately 100 patients have completed 1 year of follow-up • Interim Analysis 2: eGFR slope will be assessed when approximately 200 patients reach 1 year. The use of change in eGFR to assess risk of graft failure in chronic active AMR is based on prior modeling validation [4]	
**Secondary safety endpoints**	
1. TEAEs, serious TEAEs, and AESIs a. For clazakizumab, the following AESIs have been identified: LFT abnormalities, neutropenia, thrombocytopenia, hyperlipidemia, GI perforations, hypersensitivity and anaphylaxis, malignancy, and opportunistic infections 2. Viral infection monitoring for BKV, CMV, and EBV by PCR 3. Laboratory tests including LFTs, CBC, plasma lipids, hsCRP 4. Vital signs, ECGs, and physical examination 5. Incidence of antibodies to clazakizumab	
**Secondary efficacy endpoints**	
1. Incidence and time to death-censored graft loss (defined as return to dialysis, graft nephrectomy, re-transplantation, or eGFR <15 mL/min/1.73 m^2^ but excluding death from any cause) 2. Change in mean eGFR from baseline to EOT 3. Change in spot UACR from baseline to EOT 4. Change in DSA titers and MFI scores from baseline to EOT 5. Incidence of acute rejection episodes (TCMR and AMR) from baseline to EOT 6. Change in Banff lesion grading score (2015 criteria of pre-treatment to post-treatment (Week 52) kidney biopsies) [31] 7. Overall patient survival	
**Secondary PK endpoints**	
The PK endpoints for a subset of subjects who consent to participate in the PK/PD sub-study will include the following: 1. Maximum concentrations (*C*_max_, steady state *C*_max_ [*C*_max ss_]) 2. Trough concentrations (*C*_trough_, steady state *C*_trough_ [*C*_trough ss_]) 3. Area under the concentration-time curve at steady state (AUC_0-tau ss_) 4. Time of maximum concentration (*T*_max_, steady state *T*_max_ [*T*_max ss_])	

*AESI* adverse event of special interest, *AMR* antibody-mediated rejection, *BKV* polyoma BK virus, *CBC* complete blood count, *CMV* cytomegalovirus, *DSA* donor-specific antibodies, *EBV* Epstein–Barr virus, *ECG* electrocardiogram, *eGFR* estimated glomerular fibrillation rate, *EOT* end of treatment, *GI* gastrointestinal, *hsCRP* high-sensitivity C-reactive protein, *LFT* liver function test, *MFI* mean fluorescence intensity, *PCR* polymerase chain reaction, *PD* pharmacodynamic, *PK* pharmacokinetic, *TCMR* T-cell-mediated rejection, *TEAE* treatment-emergent adverse event, *UACR* urine albumin-to-creatinine ratio

Secondary endpoint analyses are outlined in Table 2 and include the following: incidence and time to death-censored graft loss; change in mean eGFR from baseline to end of treatment; change in spot UACR from baseline to end of treatment; change in DSA titers and MFI scores from baseline to end of treatment; incidence of acute rejection episodes (TCMR and AMR) from baseline to end of treatment; change in Banff lesion grading score [3] of pre-treatment to post-treatment (week 52) kidney biopsies; overall patient survival; and the PK and immunogenicity of clazakizumab in kidney transplant recipients with chronic active AMR.

The following safety endpoints will be evaluated and analyzed using descriptive statistics: treatment-emergent AEs (TEAEs), serious TEAEs, and AEs of special interest (AESI) using MedDRA Version 21 or later; viral infection monitoring for polyoma BK virus (BKV), CMV, and Epstein–Barr virus (EBV) by polymerase chain reaction (PCR); laboratory tests including liver function tests (LFTs), complete blood count (CBC), plasma lipids, and hsCRP; vital signs, electrocardiograms (ECGs), and physical examination; and incidence of anti-clazakizumab antibodies. Two interim safety reviews will also be conducted once approximately 50 and 100 patients have received at least one dose of treatment. These safety analyses overlap with the planned interim efficacy analyses; subsequent safety reviews may be conducted at the discretion of the Data and Safety Monitoring Board (DSMB).

### Dose selection, modification, and discontinuation

Dosing modification, withholding, or discontinuation of clazakizumab, and/or modification of background immunosuppression (such as MMF) will be required for abnormal LFTs; neutrophil and platelet counts; viral infection with BKV, CMV, and EBV; and hypogammaglobulinemia, according to defined criteria. Suggested dose reductions based on viral load are stated in the protocol. If treatment is withheld for ≥3 consecutive doses because of an AE, the medical monitor should be consulted to consider permanently discontinuing the investigational drug. Patients who discontinue treatment because of an AE and who have not reached an endpoint of graft loss or death will continue in the study and continue to comply with the protocol.

### Blinding

To maintain blinding, an unblinded pharmacist/qualified personnel at each investigational site will dispense either clazakizumab or placebo into identical syringes, according to each subject’s randomized treatment allocation. In addition, the DSMB will conduct all interim analyses.

If an AE occurs and requires the subject’s investigational product allocation to be revealed to manage the subject’s condition and/or for regulatory reporting of a suspected unexpected serious adverse reaction, the blinding code for that subject may be broken by the investigator or the sponsor’s medical monitor and the treatment identified.

### Statistical analyses

The primary efficacy endpoint will be analyzed using the Kaplan–Meier method to compare the median time-to-event between each treatment arm. Incidence and hazard ratios will also be presented. To assess the robustness of the primary efficacy analysis, the primary efficacy variable will be repeated in sensitivity analyses using the per-protocol set. The analysis of the primary endpoint when at least 221 all-cause graft loss events have occurred assumes (a) a rate of all-cause graft survival of 23% for the placebo arm over a period of approximately 5 years; (b) at least 221 graft loss events will be required to provide 80% power to detect a 31% reduction in the relative risk of graft loss compared with the placebo group, assuming clazakizumab can reduce the slope of eGFR decline by 50%; and (c) a relative risk reduction of 31% would improve the 5-year graft survival rate from 23 to 37%. Approximately 350 subjects will be enrolled to allow for 10% loss to follow-up or withdrawals.

The interim efficacy endpoint of eGFR slope at 1 year will be analyzed using a “mixed model repeated measures” approach. The model will include terms for treatment, stratification factors, baseline eGFR, and other pre-defined covariates.

For those in the PK/PD sub-study, data will be listed and summarized by nominal timepoint. The PK parameters will be derived by non-compartmental analysis using WinNonLin® version 5.2 (or higher) and will be summarized descriptively. Safety endpoints will be presented using descriptive statistics.

The ITT population will consist of all subjects who have received at least one dose of the drug and had a baseline and at least one post-baseline assessment. The per-protocol set will consist of all subjects who satisfy the ITT criteria and have no major protocol deviations that impact the primary efficacy endpoint for the final or interim analysis.

### Sample size and power

The sample size was based on data obtained from the modeling exercise [4]. Assuming a rate of all-cause allograft survival of 23% for the placebo arm over a period of approximately 60 months, at least 221 allograft loss events (expected to accrue in 316 subjects) will be required to provide 80% power (two-sided alpha of 0.05) to detect a 31% reduction in the relative risk of allograft loss in the clazakizumab group compared with the placebo group, assuming clazakizumab can reduce the slope of eGFR decline by 50%. A relative risk reduction of 31% would improve the 5-year graft survival rate from 23 to 37%. Approximately 350 subjects (175 per group) will be enrolled to allow for 10% loss to follow-up and withdrawals. Strategies to improve enrollment include both decreasing the screen failure rate and increasing the number of active sites for recruitment. This will be achieved through focused site outreach and retraining, as well as increasing the number of countries and sites to allow for more international recruitment of subjects into the study.

### Status

International recruitment is ongoing across sites in North America, Europe, Asia, and Australia. The COVID-19 pandemic has had an impact on recruitment, given the study-wide shutdown of site activity during the pandemic in March 2020. In light of this, and in order to minimize the risk to study patients, the protocol was revised to include COVID-19 PCR testing at screening, and an additional exclusion criterion was added to exclude subjects with an active COVID-19 infection. There is an option for home health visits, and patient concierge services are currently being explored with the aim of reducing the burden and improving retention of study subjects.

### Steering committee

- DSA Central Laboratory—Denise Pochinco and Peter Nickerson: confirmed patient eligibility to ensure DSA were present.
- Central Renal Pathology—Michael Mengel: confirmed patient eligibility to ensure chronic active AMR was present and the primary etiology for kidney dysfunction.
- Independent Data Monitoring Committee/DSMB—Jeremy Chapman (chair); Bruce Kaplan, Alain Jardine, Michael Ison, Simon Day (all members), and Hallvard Holdaas (former member): independent oversight of the trial to ensure trial integrity and patient safety.
- Steering Committee—Peter Nickerson (Chair); Roslyn Mannon, Arjang Djamali, Deepali Kumar, Teun van Gelder, Georg Boehmig, and Steve Chadban (all members): to provide study execution and oversight and to serve as patient experts during trial execution.

### Dissemination plan

Trial findings will be disseminated at national and international scientific meetings, by publication in a scientific journal, and through submission of the results to trial registration databases. Authorship for all trial publications will be based on criteria formulated by the International Committee of Medical Journal Editors, available at www.icmje.org.

Additionally, results will be disseminated to study patients, study staff, clinicians, and patient groups via direct approaches, and a variety of traditional and electronic media.

## Discussion

The IMAGINE study represents the first Phase 3 clinical trial investigating the safety and efficacy of clazakizumab in kidney transplant recipients with chronic active AMR, and the largest placebo-controlled trial in this patient population. As there are no approved drugs for the treatment of chronic active AMR, the findings of this study will be fundamental in helping to address the unmet need for novel therapies, given that AMR is a major cause of graft loss and burden of disease.

The primary efficacy endpoint in the IMAGINE study is time to all-cause composite graft loss, defined as a sustained return to dialysis, graft nephrectomy, re-transplantation, a sustained eGFR <15 mL/min/1.73 m^2^, or death from any cause. One of the challenges in evaluating late-stage transplant rejection is the need for substantial follow-up periods; consequently, eGFR slope has been included in this study as a reasonably likely surrogate endpoint for assessing risk of all-cause graft loss, based on data in both chronic kidney disease (CKD) and chronic active AMR [4, 35–38].

Surrogate endpoints are important for kidney transplantation trial design as they enable long-term clinical outcomes to be predicted at earlier time points [39]. As a key feature of a surrogate endpoint, decline in eGFR reflects the clinical consequence of chronic active AMR and is routinely relied upon in clinical practice to evaluate kidney function [4, 36]. eGFR slope has been evaluated in CKD; it has already been used as a surrogate for deterioration in kidney function and for defining kidney failure [35] and has been shown to be associated with clinical outcome [38] and CKD progression [37]. eGFR declines of 40 and 30% have also been accepted by the Food and Drug Administration (FDA) as a basis for drug approval of therapies intended to treat common and rare CKDs, respectively [38]. Recent modeling data in patients with active or chronic active AMR have demonstrated that both eGFR at diagnosis and change in renal function (eGFR slope) at 1 year post-diagnosis were independently associated with death-censored graft loss [4]: a 50% improvement in the slope of monthly eGFR decline at 12 months predicted an event-free survival rate of 44% at 5 years. These data support the potential utility of eGFR as a surrogate endpoint for assessing risk of all-cause graft loss (including both patient death and graft loss) in pivotal clinical trials for late AMR therapies. Routine site monitoring and monitoring oversight procedures are established. This includes the Clinical Research Associate, who will ensure that sites have the current laboratory supplies for appropriate testing; the Clinical Trial Educator who is responsible for targeted site retraining; and the unblinded teams at both CSL Behring and ICON who manage the drug accountability requirements.

In addition to the incorporation of eGFR slope at 1 year as a reasonably likely surrogate endpoint for graft loss, the design of the IMAGINE study has a number of other unique features, which are summarized in Table 3. Crucially, this study is the only Phase 3 trial underway in chronic active AMR after kidney transplantation, and the SC route and Q4W administration open the possibility for allowing self-administration at home for these patients, removing the need for infusion centers for this therapeutic intervention. This also increases the chance of long-term adherence to treatment.

**Table 3:** Unique features of the IMAGINE study

The design of the IMAGINE study has a number of unique features: • The study includes prognostic biomarker enrichment—Banff (cg) >0 with HLA DSA. • The study uniquely incorporates a reasonably likely surrogate endpoint for all-cause graft loss: eGFR slope at 1 year, which may accelerate the approval of a novel therapy for patients at risk of graft loss. Identifying an early surrogate endpoint is a major challenge and this represents a first in kidney transplantation [40] • IMAGINE is the only Phase 3 trial underway in chronic active AMR after kidney transplantation. • Crucially, the study follows patients through to the clinical endpoint of all-cause graft loss to fully evaluate the clinical potential of clazakizumab. • Sample size re-estimation when approximately 100 patients have completed 1 year of follow-up will ensure the surrogate endpoint trial is appropriately powered. • IMAGINE has been accepted by the FDA as a single study for approval, which is unusual for the transplant therapy area. • The uniqueness of the SC route and Q4W administration opens the possibility for delivering home care for these patients, removing the need for infusion centers for this therapeutic intervention. • Treatment with clazakizumab is a sustained regime, akin to treatments for autoimmune diseases, rather than the typical short course nature of current AMR and TCMR regimes.	

*AMR* antibody-mediated rejection, *cg* chronic glomerulopathy, *DSA* donor-specific antibodies, *eGFR* estimated glomerular fibrillation rate, *FDA* Food and Drug Administration, *HLA* human leukocyte antigen, *Q4W* once every 4 weeks, *SC* subcutaneous, *TCMR* T-cell-mediated rejection

The study also comes with potential limitations and challenges. The selection criteria may limit generalizability to the wider chronic AMR patient population that may include patients with histologic chronic active AMR without HLA DSA, nephrotic-range proteinuria or rapidly declining kidney function. The inclusion and exclusion criteria aim to include as homogenous a chronic active AMR population as possible and to exclude patients with advanced disease that is likely to be irreversible regardless of therapy. In addition, the risk of disease rebound (during treatment pauses or at study completion) should be monitored closely.

The results of the IMAGINE study will be informative for the management of kidney transplant recipients with chronic active AMR. The current standard of care is based on inconclusive data from small, poorly controlled studies, so these therapies have had mixed results [41–45]: many centers use a combination of apheresis (PLEX, immunoadsorption [used in some European countries]), and IVIg) +/− rituximab, based on low-level evidence of effectiveness. Recently, bortezomib, which targets alloantibody-producing plasma cells, has been investigated in late AMR in the randomized, placebo-controlled BORTEJECT trial, but did not demonstrate clear evidence of a treatment effect. The difference detected between bortezomib and placebo in eGFR slope (primary endpoint) and 2-year graft survival was not significant [46]. The bortezomib-treated group also lacked improvements in histologic or molecular disease features, and DSA levels were not reduced; in addition, there was substantial toxicity [46].

The findings of the IMAGINE study will build on results from Phase 2 studies that have demonstrated promising results with clazakizumab, alongside manageable tolerability profiles. Importantly, a small Phase 2 study of clazakizumab (25 mg SC Q4W) in kidney transplant recipients with late AMR (including 90% of patients exhibiting chronic active AMR) suggests early down modulation of DSA, a significant improvement in eGFR decline compared with placebo, and a manageable safety profile consistent with other IL-6 pathway inhibitors. In this study, clazakizumab treatment was associated with a 60% improvement in eGFR slope decline during a 3-month randomized, placebo-controlled study (NCT03444103; *N*=20), and the mean slope of eGFR differed significantly between the clazakizumab and placebo groups (−0.96 versus −2.43 mL/min/1.73 m^2^/month; *P*=0.04). Patients who switched from placebo to clazakizumab in the 40-week open-label extension demonstrated significant improvement in mean eGFR slope (*P*<0.001) in comparison with the initial 3-month study phase; within 12 weeks, clazakizumab decreased DSA MFI to a median of 77% from baseline versus placebo (103%; *P*=0.035). Prolonged clazakizumab treatment also demonstrated a significant decrease in molecular AMR (*P*=0.020) and “all rejection” scores (*P*=0.037) as per kidney biopsy at 51 weeks [20]. These findings were supported by the results of a Phase 1/2 single-center study (NCT03380377; *N*=10), which showed stabilization of renal function and improvements in DSA relative intensity scores after 18 months of therapy [21]. In light of this, IMAGINE will be the first study of chronic AMR immunosuppression to use eGFR slope as a surrogate endpoint to allow early prediction of all-cause graft loss.

In conclusion, chronic active AMR is a major cause of graft loss, with no effective treatments, and there is a lack of clinical data to support treatment decisions. The Phase 3, multicenter, double-blind IMAGINE study will evaluate the safety and efficacy of clazakizumab for the treatment of chronic active AMR in kidney transplant recipients; if successful, this pivotal trial has the potential to provide the first approved therapy for these patients. This trial includes prognostic biomarker enrichment (Banff cg >0 with HLA DSA), and uniquely incorporates a surrogate endpoint for graft loss (eGFR slope at 1 year), which may accelerate the approval of a novel therapy for patients at increased risk for graft loss.

## Trial status

### Protocol version

Protocol version number: Amendment 6

Protocol date: February 4, 2021

## Supplementary Information

**Additional file 1.**

## Data Availability

CSL will own the final datasets. One contract research organization (CRO), ICON, is generating the SDTM dataset. Another CRO, Parexel, is generating the ADaM datasets. These datasets will be transferred to CSL Behring, and no one will have access to the datasets until requested or released by CSL Behring. Throughout the study the data are processed according to ICON’s Standard Operating Procedures, which include monitoring and storing of the case-report forms; ongoing data management and medical reviews, which are focused on the completeness and coherency of the case-report forms from a clinical and safety data perspective, respectively; and established system-generated alerts and resolution processes to ensure that data are input correctly and that there are no missing data.

## Abbreviations

ACEI: Angiotensin-converting enzyme inhibitor
AE: Adverse event
AESI: Adverse event of special interest
AMR: Antibody-mediated rejection
ARB: Angiotensin II receptor blocker
BKV: polyoma BK virus
CBC: Complete blood count
cg: Chronic glomerulopathy
CKD: Chronic kidney disease
CMV: Cytomegalovirus
DSA: Donor-specific antibody/antibodies
DSMB: Data and Safety Monitoring Board
EBV: Epstein–Barr virus
ECG: Electrocardiogram
eGFR: Estimated glomerular filtration
EOT: End of treatment
EQ-5D-5L: EuroQOL-5 dimensions, 5 level questionnaire
FACIT: Functional Assessment of Chronic Illness Therapy
FDA: Food and Drug Administration
GI: Gastrointestinal
HLA: Human leukocyte antigen
hsCRP: High-sensitivity C-reactive protein
ICF: Informed consent form
IEC: Independent ethics committee
IgG: Immunoglobulin G
IL-6: Interleukin-6
IL-6R: IL-6 receptor(s)
IRB: Institutional review board
ITT: Intention-to-treat
IVIg: Intravenous immunoglobulin
KDQoL-36: Kidney Disease Quality of Life- 36-Item Short-Form Survey
LFT: Liver function test
MFI: Mean fluorescence intensity
MMF: Mycophenolate mofetil
PCR: Polymerase chain reaction
PD: Pharmacodynamic(s)
PK: Pharmacokinetic(s)
PLEX: Plasma exchange
Q4W: Once every 4 weeks
SC: Subcutaneous
TEAE: Treatment-emergent adverse event
TCMR: T-cell-mediated rejection
TG: Transplant glomerulopathy
T_reg_ cell: Regulatory T-cell
UACR: Urine albumin-to-creatinine ratio
wk: Week
